# Impact of COVID-19 Vaccinations on Menstrual Bleeding

**DOI:** 10.7759/cureus.47360

**Published:** 2023-10-20

**Authors:** Mortadah Alsalman, Fatimah Alhubail, Fidaa Bin obaid, Ibtisam Algouf, Bayan Alomair, Sara Albunyan, Lina AlMudayris, Zaenb Alsalman, Abdul Sattar Khan

**Affiliations:** 1 Department of Medicine, College of Medicine, King Faisal University, Al-Hofuf, SAU; 2 Department of Family and Community Medicine, College of Medicine, King Faisal University, Al-Hofuf, SAU

**Keywords:** bleeding, menstrual abnormality, vaccine, sars-cov-2, covid-19

## Abstract

Introduction: Vaccination has a fundamental role in protecting against and modifying the severity of several infectious diseases, including COVID-19. Several immune and non-immune adverse events have been reported post-COVID-19 vaccine. The aim of this study was to assess the effect of the COVID-19 vaccine on women’s menstrual bleeding.

Methods: A cross-sectional study was conducted among 399 vaccinated women in the Eastern Province of Saudi Arabia. Data were collected using a direct interview-based questionnaire including four sections.

Results: A total of 399 women were recruited, with a mean age of 25.54 ± 6.177 years. More than half (53.9%) of the participants reported post-vaccination menstrual bleeding abnormality in terms of a heavy or irregular cycle. Out of the total number, 40 (21.4%) women reported having heavy menstrual flow, and 67 (16.8%) had non-menstrual bleeding after receiving the COVID-19 vaccine. Fully vaccinated women were at a greater risk of post-vaccination menstrual bleeding abnormality (p = 0.058). However, there was no correlation between booster shot type and menstrual bleeding abnormality (p > 0.05). In addition, there was no significant association between non-menstrual bleeding and the type of booster shot, the type of the first dose, and prior history of COVID-19 infection (p > 0.05).

Conclusion: Despite vaccination being the most effective way to prevent COVID-19, it does have an impact on menstrual bleeding in terms of menorrhagia and metrorrhagia. Therefore, more studies are needed to understand the mechanism and the long-term impact of COVID-19 vaccines on the hemostatic system.

## Introduction

COVID-19 is a serious infectious disease caused by a newly discovered coronavirus called SARS-CoV-2, which was first documented in the city of Wuhan, China, at the end of 2019 [1]. The rapid and alarming global spread of this virus prompted the World Health Organization to declare a global health emergency on January 30, 2020 [1]. Countries were encouraged to undertake strict social distancing and quarantine measures to prevent virus transmission and protect public health [2]. Since the occurrence of the pandemic, international research laboratories, in collaboration with pharmaceutical and biotech companies, have been working at a fast pace to find and evaluate drugs, vaccines, and other solutions targeted at reducing hospital admissions, supporting patients in healing, and assisting recovery [3].

Four COVID-19 vaccines have been approved by the Saudi Ministry of Health. Pfizer-BioNTech (BNT162b2) and Moderna (mRNA-1273) are mRNA vaccines, whereas AstraZeneca (ChAdOx1-nCoV) and Janssen (Johnson & Johnson) are non-replicating viral vector-based vaccines [4-6]. As COVID-19 vaccination programmes have been initiated worldwide to immunise against the virus, there has been a wide range of post-vaccination local and systemic adverse events. However, serious adverse effects post-COVID-19 immunization are uncommon [7]. Up to the most recent update on August 24, 2022, the Yellow Card in the United Kingdom had recorded 51,435 cases of menstruation changes following COVID-19 vaccinations [8]. Nevertheless, the actual number is expected to be much higher, as many women may have felt uncomfortable discussing menstrual issues, may not have believed it was related to vaccination, or may have not reported it to the adverse event reporting system [9]. There are different plausible mechanisms to explain how vaccination might affect the menstrual cycle, such as vaccine-induced thrombocytopenia and post-vaccination immune activation, but the exact cause remains unknown [9].

Thus, our study aimed to look into the effect of validated COVID-19 immunizations on women’s menstrual bleeding and whether the effect extended to non-menstrual bleeding.

## Materials and methods

Study design, setting, and participants

A cross-sectional study was conducted among females in the Eastern Province of Saudi Arabia between December 2021 and December 2022 after ethical approval was obtained from King Faisal University’s Ethics Committee. Non-Saudi women, those under the age of 18, and those who failed to receive the COVID-19 immunization were all omitted.

The minimum required sample size was calculated at 397 using Cochran’s formula = Z2P(1−P)/d2; the prevalence of heavy menstrual bleeding is 65% as reported by a previous Saudi study, with the desired level of precision of 10% and a confidence interval (CI) of 95% [10].

All participants provided informed consent before proceeding to complete the survey. Data were collected using a direct interview-based questionnaire that included four sections. The first section included questions pertaining to participants’ demographic data (age, marital status, level of education, use of contraceptives, and previous COVID-19 infection). The second section elicited data related to COVID-19 vaccine (doses and type). The third part determined the participants’ perception of their menstrual bleeding before and after receiving the COVID-19 vaccine. Finally, the fourth section assesses non-menstrual bleeding (e.g., mucocutaneous bleeding and gastrointestinal (GI) bleeding).

The questionnaire was evaluated by experts through the Delphi technique and piloted in order to determine its validity and reliability (Cronbach’s alpha > 0.7) [11].

Data analysis

Collected data were analyzed using IBM SPSS Statistics for Windows, version 22 (released 2013; IBM Corp., Armonk, New York, United States). Descriptive analysis was performed using frequencies and percentages for categorical variables and mean and median values with standard deviation (SD) for continuous variables. The chi-square test was used to assess the association among different variables. P-value < 0.05 at a 95% CI was considered statistically significant.

## Results

A total of 399 women were recruited, with a mean age of 25.54 ± 6.177 years, and more than half (52%) of them belonged to the 18-23 age group. The majority of the participants (65%) were single, and less than one-fifth (14%) were using contraceptives. Moreover, nearly half (46.9%) of the participants had a history of COVID-19 infection, and almost 80% had received three doses of the COVID-19 vaccine. The remaining sociodemographic data are shown in Table 1.

**Table 1:** Sociodemographic characteristics of the participants (n=399).

Variables		n (%)
Age in years	18-23	210 (52.6)
	24-28	86 (21.6)
	29-33	49 (12.3)
	34-38	30 (7.5)
	39-43	22 (5.5)
	44-48	1(0.3)
	≥ 49	1(0.3)
Level of education	Primary	3 (0.8)
	Intermediate	2 (0.5)
	Secondary	40 (10)
	Higher	354 (88.7)
Marital Status	Single	258 (64.7)
	Married	141 (35.3)
Currently using contraceptives	Yes	55 (13.8)
	No	344 (86.2)
Confirmed previous COVID-19 infection	Yes	187 (46.9)
	No	212 (53.1)
Number of COVID-19 vaccine doses	One dose	5 (1.3)
	Two doses	76 (19)
	Three doses	317 (79.4)
	Four doses	1 (0.3)

Of the total number, 53.9% of the participants reported post-vaccination menstrual bleeding abnormality in terms of a heavy or irregular cycle. There were no significant associations between menstrual bleeding changes after receiving the vaccine and the type of booster shot, previous history of COVID-19 infection, and age group (p > 0.05). However, women who received three doses were at a greater risk of post-vaccination menstrual bleeding abnormality (p = 0.058; Table 2).

**Table 2:** Factors related to post-vaccination menstrual bleeding change.

Variables		Post-vaccination menstrual bleeding abnormality		P-value
		Yes 215 (53.9%)	No 184 (46.1%)	
Types of vaccines received by the participant	Same type of vaccine	135 (53.6%)	117 (46.4%)	0.869
	Mixed type vaccine	80 (54.4%)	67 (45.6%)	
Doses of COVID-19 vaccine	One dose	0 (0.0%)	5 (2.7%)	0.058
	Two doses	38 (17.7%)	38 (20.7%)	
	Three doses	176 (81.9%)	141 (76.6%)	
	Four doses	1 (0.5%)	0 (0.0%)	
COVID-19 infection	Yes	100 (25.1%)	87 (21.8%)	0.471
	NO	115 (28.8%)	97 (24.3%)	
Age in groups (in years)	18-23	115 (53.5%)	95 (51.6%)	0.495
	24-28	42 (19.5%)	44 (23.9%)	
	29-33	27 (12.6%)	22 (12.0%)	
	34-38	19 (8.8%)	11 (6.0%)	
	39-43	11 (5.1%)	11 (6.0%)	
	44-48	0 (0.0%)	1 (0.5%)	
	≥ 49	1 (0.5%)	0 (0.0%)	

Out of the total number, 40 (21.4%) women reported having heavy menstrual flow, and 67 (16.8%) had non-menstrual bleeding after receiving a COVID-19 vaccine. Looking at factors related to post-vaccination non-menstrual bleeding, there was no significant association between bleeding incidence and the type of booster shot, type of the first dose, or prior history of COVID-19 infection (p > 0.05; Table 3).

**Table 3:** Factors related to post-vaccination non-menstrual bleeding.

Variables		Post-vaccination non-menstrual bleeding.		P-value
		Yes 67 (16.8%)	No 332 (83.2%)	
Types of vaccines received by the participant	Same type of vaccine	41 (10.3%)	211 (52.9%)	0.407
	Mixed type of vaccine	26 (6.5%)	121 (30.3%)	
Type of 1^st^ doses of COVID-19 vaccine	Pfizer	49 (73.1%)	247 (74.4%)	0.451
	Oxford	17 (25.4%)	84 (25.3%)	
	Modrena	1 (1.5%)	1 (0.3%)	
COVID-19 infection	Yes	28 (7%)	159 (39.8%)	0.421
	NO	39 (9.8%)	173 (43.4%)	

## Discussion

Menstrual bleeding is an important indicator of women’s health, as heavier flows have been linked to an increased risk of anemia, hemodynamic instability, and poor quality of life [10,12]. During the COVID-19 pandemic, several factors impacted women’s menstrual cycles, such as infection, emotional or physical stress, and COVID-19 vaccination. Our study revealed that more than half of the COVID vaccine recipients had post-immunization menstrual bleeding abnormalities in terms of menorrhagia or metrorrhagia. Such a high figure of post-vaccination menstrual changes, ranging from 40% to 66%, has also been reported by recent studies conducted in the USA, Norway, Hungary, and the Middle East and North Africa (MENA) region [13-16]. Of note, less than one-third of the participants experienced heavy menstrual flow, which is less than the previously reported range of 40-55% [14,17]. This discrepancy in findings can be attributed to recall and selection bias.

Post-immunization menstrual abnormalities have been linked to other vaccines, for instance, the human papillomavirus (HPV) vaccine, although the exact mechanism is not yet fully understood [12,16]. According to the current study, there is no correlation between booster shot type and menstrual bleeding abnormality; however, fully vaccinated participants were at a higher risk of menstrual bleeding changes. In addition, these findings are somewhat in line with results from recent studies, which reported no difference following immunization with mRNA compared to adenovirus-vectored vaccines [16,18,19]. Furthermore, it supports the possibility that these changes are the result of post-vaccination immune activation affecting the hypothalamic-pituitary-ovarian axis, which regulates the cycle, rather than due to a specific vaccine component [12,13].

Mucocutaneous bleeding, such as GI or genitourinary bleeding, has been previously reported following vaccination, particularly with mRNA-based vaccines, and was attributed to vaccine-induced thrombocytopenia (VIT) [12,20]. Most thrombocytopenia cases following immunization were self-controlled, and only a few cases remained in the chronic form [9,21]. Similarly, our study revealed that less than one-fifth of vaccinated women experienced mild mucocutaneous bleeding, although the vaccine type had no influence on bleeding. However, the attribution of the bleeding mechanism to VIT is uncertain, as laboratory results are lacking.

Concerns about the COVID-19 vaccines' safety have been raised after recent reports of bleeding, thrombocytopenia, and thrombosis, especially among people with coagulation disorders or on anticoagulant medications [9]. Our study is the first to assess and shed light on the impact of COVID-19 vaccines on menstrual and non-menstrual bleeding in the region irrespective of the type of vaccine, although it has some limitations. It was a cross-sectional study, which increased the likelihood of recall biases and made it difficult to establish a causal relationship. In addition, this study was directed to those who had received three doses of vaccine or less. Therefore, additional effects following subsequent doses are unknown.

## Conclusions

Despite vaccination being the most effective way to prevent and mitigate the severity of COVID-19 infection, it does have an impact on menstrual bleeding in terms of menorrhagia and metrorrhagia. This paper highlights that fully vaccinated individuals are more likely to have a menstrual bleeding abnormality, irrespective of the vaccine type. This study emphasizes the importance of counseling all women about menstrual bleeding abnormalities prior to COVID-19 vaccination. In addition, there was no significant association between non-menstrual bleeding and the COVID-19 vaccine. Further studies are needed to address bleeding among vaccinated women receiving anticoagulants and the mechanism and long-term impact of COVID-19 vaccines on bleeding events.
